# Associations Between Child Maltreatment, Autonomic Regulation, and Adverse Cardiovascular Outcome in an Urban Population: The HELIUS Study

**DOI:** 10.3389/fpsyt.2020.00069

**Published:** 2020-03-17

**Authors:** Maryse J. Bakema, Mirjam van Zuiden, Didier Collard, Jasper B. Zantvoord, Susanne R. de Rooij, Leonie K. Elsenburg, Marieke B. Snijder, Karien Stronks, Bert-Jan H. van den Born, Anja Lok

**Affiliations:** ^1^Department of Psychiatry, Amsterdam UMC location AMC, University of Amsterdam, Amsterdam, Netherlands; ^2^Department of Internal and Vascular Medicine, Amsterdam UMC location AMC, University of Amsterdam, Amsterdam, Netherlands; ^3^Department of Clinical Epidemiology, Biostatistics and Bioinformatics, Amsterdam UMC location AMC, University of Amsterdam, Amsterdam, Netherlands; ^4^Department of Public Health, Amsterdam UMC location AMC, University of Amsterdam, Amsterdam, Netherlands

**Keywords:** adverse childhood experience, cardiovascular disease, autonomic regulation, heart rate variability, baroreflex sensitivity

## Abstract

**Introduction:**

A mounting body of literature emphasizes the potential negative effects of adverse childhood experiences (ACEs) on both mental and physical health throughout life, including an increased risk for developing cardiovascular disease (CVD). Since CVD is one of the leading causes of mortality and morbidity worldwide, it is of great importance to advance our understanding of the effects of on CVD. This holds both for the actual incidence and for intermediate biological pathways that may convey CVD risk, such as imbalance in autonomic nervous system regulation, resulting in a chronically heightened sympathetic activity and lowered reactivity. In a large urban, multi-ethnic population-based cohort study we investigated whether there is an association between child maltreatment, CVD incidence and autonomic regulation.

**Methods:**

Within the Health in an Urban Setting (HELIUS) study, a large, multi-ethnic population cohort study including n = 22,165 Amsterdam residents, we used logistic regression analyses to investigate the association between the number of self-reported types of child maltreatment (range 0–4), and self-reported adverse cardiovascular outcome (aCVO). Self-reported child maltreatment included emotional neglect, emotional abuse, physical abuse, and sexual abuse. Furthermore, in a subsample (n = 10,260), mean age 44.3, we investigated associations between child maltreatment, autonomic regulation, and aCVO using linear regression analyses. Both baroreflex sensitivity (BRS) and heart rate variability (HRV) were assessed as non-invasive indices of autonomic regulation.

**Results:**

The number of endorsed child maltreatment types was significantly associated with a higher aCVO risk. The association remained significant after adjustment for demographic, socioeconomic, health-behavioral, and psychological covariates (p = 0.011, odds ratio: 1.078, confidence interval: 1.018–1.142). The cumulative exposure to child maltreatment was negatively associated with BRS and HRV, but the association was no longer significant after correction for socioeconomic and demographic covariates.

**Conclusion:**

In a large, multi-ethnic urban-population cohort study we observed a positive association between number of endorsed child maltreatment types and self-reported aCVO but not autonomic regulation, over and above the effect of relevant demographic, health, and psychological factors. Future studies should examine the potential role of the dynamics of autonomic dysregulation as potential underlying biological pathways in the association between ACEs and CVD, as this could eventually facilitate the development of preventive and therapeutic strategies for CVD.

## Introduction

According to the World Health Organization (WHO), the widely used umbrella term adverse childhood experiences (ACEs) refers to some of the most intensive and frequently occurring sources of stress that happens to someone before the age of 18 that the person remembers as an adult (1), either directly (e.g., abuse and neglect), or indirectly through their living environments (e.g., parental conflict, substance abuse, or mental illness). ACEs are common and affect a substantial part of our society. Estimations from multiple studies on the prevalence of ACEs in large healthy adult populations in Western countries vary from 34 to 62% of the population having experienced one or more ACEs (2–4).

ACEs, i.e. prolonged and/or severe (traumatic) stress experiences in childhood, can have lifelong consequences for a person's health and well-being (5, 6). Cross-sectional and longitudinal studies have previously observed associations between ACEs and subsequent altered functioning of a myriad of biological systems, including the nervous, endocrine, and immune system (7, 8). This may result in impaired emotional, cognitive, physical, and social functioning, and thereby affect mental and physical health. Additionally, ACEs have been described to indirectly negatively impact mental and physical health through associations with increased maladaptive lifestyle and behavior (such as smoking, alcohol use, physical inactivity, and an unhealthy diet) (5, 9–14); decreased access to life opportunities; and lack of economic stability, although the directionality of causality in these associations is difficult to establish (15). Thus, given the evidence of their negative long-term impact on public health, ACEs are of great concern.

One of the unfavorable health outcomes associated with ACEs is an increased risk for developing cardiovascular disease (CVD), one of the leading causes of mortality and morbidity worldwide (16). ACEs have been associated with a significantly increased risk of both self-reported and objectively verified CVD in several studies, including general population cohorts and CVD case–control studies [for systematic review see (17)].

The pathophysiological pathway through which ACEs increase the risk of CVD is likely to be multifactorial, with autonomic nervous dysregulation as one of the potential underlying physiological mechanisms leading to CVD risk (7). Both baroreflex sensitivity (BRS) and heart rate variability (HRV) are non-invasive indices of autonomic regulation. HRV, the beat-to-beat variation in the heartrate over time, is a reliable index of autonomic nervous regulation (18). Decreased HRV indicates an inability to reduce sympathetic activation of the heart (19). This makes the heart vulnerable to arrhythmia and sudden death and also accelerates development of atherosclerotic coronary artery disease (20, 21). Reduced HRV has been established as a prognostic risk factor for CVD. For example, lower HRV has been shown to be an independent predictor of cardiac events and mortality in both healthy individuals and patients with a history of myocardial infarction (19, 22). Additionally, BRS is the magnitude of change in heart rate interval in response to a change in systolic blood pressure (SBP) (17). The baroreflex is important for hemodynamic stability and for cardio protection. It has however repeatedly been demonstrated that a decrease in BRS is a prognostic factor for CVD and arrhythmic events post-myocardial infarction [MI (19)].

Current models assume that ACEs increase CVD risk by altering long-term patterns of autonomic regulation by disturbing the sympathetic and parasympathetic balance (7, 23), characterized by enduring higher sympathetic activity and lowered parasympathetic activity. Multiple studies have shown associations between ACEs and HRV, generally finding lower HRV to be associated with ACEs, but not all findings in non-clinical, depressed and adolescent samples have been consistent (24–28). The association between ACEs and BRS has not been examined before.

In this study, we aimed to investigate whether exposure to child maltreatment increases the risk for CVD, i.e. self-reported adverse cardiovascular outcomes (aCVO), and autonomic regulation (as measured with HRV and BRS) in the general adult population. The focus of the current study is on child maltreatment as this concerns a relatively common and directly experienced type of ACE, which may occur in the form of physical, sexual, and psychological abuse, and emotional neglect, and has been previously observed to have pervasive consequences for subsequent health and well-being of affected individuals throughout life. We used data from the Health in an Urban Setting (HELIUS) study, which is a large representative urban cohort study, N = 22,615, among several ethnic groups living in Amsterdam, the Netherlands. The design of the HELIUS study guarantees a heterogeneous cross-section of the general Western population, generalizable on demographic factors such as socioeconomic status (SES), educational level, and ethnicity (29, 30).

## Methods

The HELIUS study is a large cohort study carried out in Amsterdam, the Netherlands. The HELIUS study mainly focuses on three disease categories: CVD, mental health, and infectious diseases. Between 2011 and 2015, baseline HELIUS data were collected among Amsterdam residents of South-Asian Surinamese, African Surinamese, Turkish, Moroccan, Ghanaian, and Dutch origin. Persons were randomly sampled, by ethnic origin, *via* the municipality register of Amsterdam and received an invitation to take part in the study. Contact could be made with 55% of selected invitees, of which approximately 50% responded and agreed to participate, leading to a total response rate of 28%. Previous non-response analyses revealed only minor differences in socioeconomic characteristics between invited individuals agreeing to participate, invited individuals not participating, and non-invited eligible individuals, indicating that the HELIUS cohort is representative for the investigated ethnic groups living in Amsterdam [for details see (29, 30). Data were collected through an extensive questionnaire and a physical examination that included the collection of biological samples. The Institutional Review Board of the Amsterdam UMC location AMC approved the study's protocols and all participants gave written informed consent. For the current analyses, we used data of n = 22,165 participants for whom data from the questionnaire regarding child maltreatment and aCVO as well as the physical examination were available.

### Measures

#### Child Maltreatment

The occurrence of child maltreatment was assessed using a questionnaire based on the NEMESIS Trauma questionnaire (31). It contains four items, reflecting specific types of maltreatment experienced before the age of 16: emotional neglect (ignored or unsupported), physical abuse (kicked, hit, bitten, or hurt), emotional abuse (yelled at, insulted, or threatened), and sexual abuse (any unwanted sexual experience). A Likert scale was used to answer these questions (never–once–sometimes–often–would rather not say). Maltreatment types were scored as endorsed when experienced sometimes or often, except for sexual abuse which was scored as endorsed when experienced at least once. Finally, the total number of maltreatment types endorsed was calculated (range: 0–4) (see below for details on imputation strategy in case of missing data).

#### aCVO

History of CVD, i.e. aCVO, was based on three self-report items in the HELIUS questionnaire: 1) Have you ever had a heart attack (MI)? 2) Have you ever had a stroke? 3) Have you ever had a dotter procedure (angioplasty) or a bypass operation on your heart or legs? The scale was dichotomized to 0 (no reported CVD) and 1 (at least one item endorsed). Three hundred seventeen participants were excluded due to missing data on self-reported CVD.

#### Autonomic Regulation

Electrocardiogram (ECG) measurements were performed in all participants (MAC 1600 System, GE Healthcare). Due to logistic constraints, only a subset of n = 13,726 participants was subjected to continuous blood pressure (BP) measurement by finger plethysmography using the Nexfin device (BMEYE, Amsterdam). BP measurements were obtained for 3–5 min in the supine position after 10-min rest. Automated analysis of the hemodynamic data was performed using custom-written software in Matlab (R2018b, The MathWorks, Inc. Natick) as described elsewhere (32). In brief, to remove noise and possible ectopic beats, the beat-to-beat dataset was filtered using a local moving median filter with a length of nine beats. Beats which duration diverted more than 25% of the mean interbeat interval (IBI), the duration between successive heartbeats, from the local median IBI were removed. Participants with no detected sinus rhythm on the ECG or more than 20% removed beats were excluded (32). This yielded n = 10,260 participants with available HRV and BRS data. We observed several small but statistically significant differences between included and non-included participants (see **Supplementary Table 1**). Compared to non-included participants, those included were more commonly males (45.8% versus 39.1%) and on average 5 months younger. Also, included participants had a somewhat higher BMI, but reported a lower number of maltreatment types experienced and lower prevalence of CVD.

#### HRV

HRV was determined by calculation of the mean IBI, the square root of the mean squared successive differences between adjacent normal-to-normal (NN) intervals (RMSSD) and the standard deviation of NN intervals (SDNN), following international guidelines (33). For the analyses, both RMSSD and SDNN (in ms) were included, as they both measure different aspects of HRV (34, 35). RMSSD mostly reflects the parasympathetic system, while SDNN reflects all the cyclic components responsible for HRV in the period of recording with the influence of the sympathetic system and all other influential systems on the heart rate included (33, 35, 36).

#### BRS

Estimates of BRS were obtained from beat-to-beat changes in SBP and heart rate, using the xBRS method. First, the change in heart rate in response to change in BP was assessed, using a cross-correlation method over a sliding 10-s window with various delay compensations (37, 38). Second, for each segment with a significant positive cross-correlation, xBRS was obtained by dividing the standard deviation of the IBI by the standard deviation of the SBP. The overall xBRS (in ms/mm Hg) is the geometric mean of all obtained xBRS values per segment with a significant positive cross-correlation.

#### Covariates

Socioeconomic and demographic variables included were age, sex, ethnicity, and educational level. Ethnicity was divided into seven groups: Dutch, South-Asian Surinamese, African Surinamese, Ghanaian, Turkish, Moroccan, and “other.” This latter group consists of participants with a Javanese-Surinamese origin, other/unknown Surinamese origin, or other/unknown ethnic origin, which were combined due to relatively small sample size (2.5% of total sample size). In all analyses, ethnic minority groups were compared to the Dutch ethnicity. Educational level was divided into two categories: 1) no or lower education (no schooling, elementary schooling only, lower vocational schooling, or lower secondary schooling) and 2) intermediate or higher education (intermediate vocational schooling, intermediate/higher secondary schooling, higher vocational schooling, or university) (30).

Furthermore, the following health behaviors and anthropometric measurements were taken into account: current smoking (yes/no), alcohol intake in past 12 months (yes/no), physical activity (reaching the international goal of 30 min per day on at least 5 days per week: yes/no), and body mass index (BMI) (measured during physical examination, kg/m^2^), as well as current stress (negative life events in the last 12 months: yes or no). A broad range of studies have consistently shown associations between ACEs and higher risk of adult smoking (12), obesity (15), physical inactivity (39) and heavy alcohol use (5), and increased experienced stressors in adulthood, as well as associations between these variables and aCVO risk, and thus these factors represent potential mediating pathways in the child maltreatment–aCVO association.

Because of the expected influence of use of antihypertensive medication on both HRV and BRS, use of antihypertensives (dichotomous) was also corrected for in analyses including autonomic measures.

### Statistical Analysis

For the data analyses in this study, IBM SPSS statistics version 25.0 was used. A two-sided p-value of <0.05 was considered significant. Because of the high percentage of missing values in child maltreatment data (range: 4.4% for Dutch participants, up to 12% for Ghanaian participants), multiple imputation was performed to avoid underestimation of the prevalence of child maltreatment and thereby biased results on the probable association between child maltreatment and our outcomes. Multiple imputation is an effective method for dealing with missing data (40), and is becoming increasingly common, also specifically for studies on ACEs (41, 42). As missingness (item either left blank or scored “would rather not say”) correlated with the large majority of variables, the missing data for the four child maltreatment items were imputed in SPSS using Markov Chain Monte Carlo imputation with fully conditional specification with auxiliary variables (main effects and two-way interactions among categorical predictors) and predictive mean matching. All variables used for our final analysis were included as auxiliary variables (40). Eventually 30 imputations were needed to obtain adequate imputation efficiency. All reported findings concern the pooled results from these 30 imputed datasets.

We first investigated whether child maltreatment were significantly associated with aCVO, using binomial logistic regression analyses. Secondly, we investigated whether child maltreatment were significantly associated with the three autonomic measures: RMSSD, SDNN, and BRS, using three separate sets of linear regression analyses. Third, we investigated whether the three autonomic measures were associated with aCVO, using three separate sets of binomial logistic regression analyses. Prior to each regression analysis, it was confirmed that relevant statistical assumptions were met.

In each regression analysis, adjustment for covariates was performed in a stepwise manner. First, we assessed a model without any covariates added. Second, we added a block of covariates regarding demographic and socioeconomic variables that may potentially confound the association between child maltreatment and CVD. Third, a block of covariates regarding health-behavioral characteristics and chronic stress was added. This latter block represents potential mediating factors in the child maltreatment–aCVO association. It was included in the regression models as we aimed to examine whether there was an additional effect of child maltreatment on CVD over and above these previously described mediating pathways. Additionally, for the models containing autonomic measures only, antihypertensive medication use added as additional covariate in a separate block, before adding the other covariate blocks. Furthermore, a sensitivity analysis was performed to adjust for the potential influential effect of already experienced aCVO on the relationship between child maltreatment and autonomic regulation, excluding all participants reporting aCVO.

To assess whether moderation effects between ACEs and age or gender should be considered in addition to their main effects, regression analyses on associations between child maltreatment and aCVO, HRV, and BRS were additionally performed including the interaction effects between number of endorsed child maltreatment types and gender or age group respectively. For this purpose, the age variable was categorized into four categories containing equal percentages of participants (i.e. quartiles): 18–32, 33–45, 46–53, and 54–70. The first three categories were compared to the eldest category, because aCVO was expected to be highest within that group. Sex and age did not significantly moderate the effect of child maltreatment on BRS or HRV, nor the effect of child maltreatment on aCVO (**Supplementary Tables 5A, B**), and therefore we considered only main effects of age and gender in the results.

## Results

Sample characteristics per ethnic group are shown in **Table 1**. The average age of all participants was 44.3 (SD = 13.2) years, with 57.8% women. Overall, 7676 participants (34.6%) experienced any type of child maltreatment, and 1135 participants (5.2%) reported a history of aCVO.

**Table 1:** Baseline characteristics of the study population.

	Total (N = 22,165)	Dutch (N = 4564)	South-Asian Surinamese (N = 3043)	African Surinamese (N = 4151)	Ghanaian (N = 2339)	Turkish (N = 3614)	Maroccan (N = 3906)	Other (N = 548)
**Sociodemographics**								
Age	44.3 ± 13.2	46.2 ± 14.1	45.5 ± 13.4	47.9 ± 12.5	44.7 ± 11.2	40.4 ± 12.2	40.5 ± 12.9	47.5 ± 12.5
Female	12,810 (57.8%)	2475 (54.2%)	1672 (54.9%)	2535 (61.1%)	1434 (61.3%)	1980 (54.8%)	2392 (61.2%)	322 (58.8%)
Educational level								
No–lower	9679 (44.1%)	796 (17.5%)	1447 (47.8%)	1708 (41.5%)	1577 (68.7%)	2024 (56.6%)	1899 (49.1%)	228 (42.5%)
intermediate–higher	12,279 (55.9%)	3743 (82.5%)	1580 (52.2%)	2407 (58.5%)	720 (31.3%)	1552 (43.4%)	1969 (50.9%)	308 (57.5%)
**Health behavior**								
Smoking	5302 (24.0%)	1129 (24.8%)	861 (28.4%)	1309 (31.7%)	104 (4.5%)	1240 (34.6%)	525 (13.5%)	134 (24.8%)
Drinking alcohol	11,221 (50.9%)	4151 (91.1%)	1708 (56.4%)	2826 (68.6%)	1101 (47.6%)	813 (22.7%)	286 (7.4%)	336 (62.6%)
BMI	27.1 ± 5.3	24.8 ± 4.2	26.3 ± 4.8	27.8 ± 5.5	28.4 ± 5.0	28.6 ± 5.7	27.6 ± 5.2	26.7 ± 5.0
**Child maltreatment**								
Any type	7676 (34.6%)	1689 (37.0%)	1132 (37.2%)	1645 (39.6%)	688 (29.4%)	1231 (34.1%)	1066 (27.3%)	225 (41.1%)
Emotional neglect	5542 (25.0%)	1287 (28.2%)	828 (27.2%)	1070 (25.8%)	412 (17.6%)	1017 (28.1%)	763 (19.5%)	165 (30.1%)
Emotional abuse	3615 (16.3%)	734 (16.1%)	609 (20.0%)	820 (19.8%)	278 (11.9%)	514 (14.2%)	538 (13.8%)	122 (22.3%)
Physical abuse	3640 (16.4%)	443 (9.7%)	626 (20.6%)	969 (23.3%)	427 (18.3%)	505 (14.0%)	556 (14.2%)	114 (20.8%)
Sexual abuse	1871 (8.4%)	531 (11.6%)	232 (7.6%)	585 (14.1%)	156 (6.7%)	116 (3.2%)	181 (4.6%)	70 (12.8%)
Number of types experienced								
0	14,489 (65.4%)	2875 (63.0%)	1911 (62.8%)	2506 (60.4%)	1651 (70.6%)	2383 (65.9%)	2840 (72.7%)	323 (58.9%)
1	3464 (15.6%)	856 (18.8%)	455 (15.0%)	644 (15.5%)	333 (14.3%)	628 (17.4%)	461 (11.8%)	87 (15.9%)
2	2000 (9%)	455 (9.9%)	279 (9.2%)	408 (9.8%)	169 (7.2%)	327 (9.0%)	304 (7.8%)	58 (10.6%)
3	1640 (7.4%)	282 (6.2%)	307 (10.1%)	389 (9.4%)	141 (6.0%)	233 (6.5%)	236 (6.0%)	52 (9.5%)
4	572 (2.6%)	96 (2.1%)	90 (2.9%)	204 (4.9%)	45 (1.9%)	43 (1.2%)	65 (1.7%)	28 (5.1%)
**CVD**	1135 (5.2%)	167 (3.7%)	271 (9%)	216 (5.3%)	101 (4.5%)	233 (6.6%)	126 (3.3%)	21 (3.9%)
**Autonomic regulation**								
N	10,260	2045	1341	2079	1251	1729	1815	0
BRS	13.43 ± 9.07	14.98 ± 10.55	12.51 ± 8.31	12.96 ± 8.44	12.63 ± 8.82	12.69 ± 8.39	14.16 ± 9.61	–
RMSSD	46.73 ± 29.46	49.70 ± 32.43	43.63 ± 30.09	46.68 ± 29.60	47.35 ± 26.97	44.13 ± 27.26	47.77 ± 28.7	–
SDNN	52.41 ± 27.82	58.50 ± 31.16	51.43 ± 28.97	50.73 ± 26.84	49.63 ± 26.75	50.71 ± 26.10	51.72 ± 25.31	–

Baseline characteristics per ethnic group, described as means and standard deviations for continuous variables, and frequency and percentage for categorical variables. CVD, cardiovascular disease; BRS, baroreflex sensitivity; RMSSD, a parameter reflecting heart rate variability calculated as the square root of the mean squared successive differences between adjacent normal-to-normal interbeat intervals; SDNN, a parameter reflecting heart rate variability calculated as the standard deviation of normal-to-normal interbeat intervals; BMI, body mass index.

### Association Between Child Maltreatment and aCVO

A higher number of endorsed ACE types was significantly associated with a 1.1 times higher risk for aCVO, as shown in **Table 2**. After correction for all covariates, this association remained significant, with each additional child maltreatment type endorsed increasing the odds of aCVO by 7.8% [95% confidence interval (CI): 1.05–1.17, p = 0.011]. The corresponding table is shown in **Supplementary Table 2**.

**Table 2:** Logistic regression analyses on the association between child maltreatment and adverse cardiovascular outcome (aCVO).

	R^2^	β		Odds ratio	95% Confidence interval	p
Model 1	0.002	0.101		1.106	[1.049, 1.167]	<0.001
Model 2*	0.150	0.108		1.114	[1.054, 1.178]	<0.001
Model 3*^†^	0.159	0.096		1.100	[1.039, 1.165]	0.001
Model 4*^†‡^	0.164	0.075		1.078	[1.018, 1.142]	0.011

Each model shows the regression results of child maltreatment–number of types endorsed.

*Adjusted for sociodemographic covariates (sex, age, education, and ethnicity).

^†^Adjusted for health-behavioral covariates (smoking, alcohol, body mass index, physical activity).

^‡^Adjusted for psychological covariate (current stress).

### Association Between Child Maltreatment and Autonomic Regulation

In the models without covariates, the number of endorsed ACE types was significantly associated with RMSSD and BRS, but not SDNN (**Table 3**). An increasing number of child maltreatment types endorsed was significantly associated with lower BRS and lower RMSSD. These associations were not affected by additionally adjusting for antihypertensive medication use but were no longer present after adding the sociodemographic covariates to the model (see **Supplementary Tables 3A, B**). This was not affected by the final step of adding the covariates concerning health-behavioral characteristics and current stress to the model. Results remained unchanged after excluding participants reporting previous aCVO (**Supplementary Table 3C**).

**Table 3:** Multiple linear regression analyses on association between child maltreatment and xBRS and heart rate variability.

	R^2^	β	95% Confidence Interval	p
**xBRS**				
Model 1	0.001	−0.016	[−0.028, −0.005]	0.006
Model 2*	0.068	−0.017	[−0.028, −0.006]	0.003
Model 3*^†^	0.370	−0.002	[−0.009, 0.010]	0.907
Model 4*^†‡^	0.395	−0.001	[−0.007, 0.008]	0.981
**RMSSD**				
Model 1	0.001	−0.015	[−0.025, −0.004]	0.007
Model 2*	0.031	−0.015	[−0.026, −0.005]	0.004
Model 3*^†^	0.220	−0.003	[−0.013, 0.006]	0.530
Model 4*^†‡^	0.233	−0.007	[−0.016, 0.003]	0.169
**SDNN**				
Model 1	0.000	−0.006	[−0.014,0.003]	0.196
Model 2*	0.041	−0.006	[−0.015,0.002]	0.148
Model 3^†^	0.184	−0.003	[−0.005,−0.011]	0.760
Model 4*^†‡^	0.196	0.001	[−0.007, 0.009]	0.747

N = 10,260. Each model shows the regression results of child maltreatment-number of types endorsed on xBRS/RMSSD and SDNN.

*Adjusted for antihypertensive medication.

^†^Adjusted for sociodemographic covariates (sex, age, education, and ethnicity).

^‡^Adjusted for health-behavioral and psychological covariates (smoking, alcohol, body mass index, physical activity, current stress).

xBRS, baroreflex sensitivity; RMSSD, a parameter reflecting heart rate variability calculated as the square root of the mean squared successive differences between adjacent normal-to-normal interbeat intervals; SDNN, a parameter reflecting heart rate variability calculated as the standard deviation of normal-to-normal interbeat intervals.

### Associations Between Autonomic Regulation and aCVO

The main effects of BRS, RMSSD, SDNN, on aCVO were all significant, also after adjustment for antihypertensive medication use. Lower BRS, RMSSD, and SDNN were associated with increased odds for aCVO (**Table 4**). For both HRV parameters, the association was no longer significant after adding socioeconomic covariates to the model (see **Supplementary Table 4B**). For BRS, the association remained significant after adjusting for sociodemographic covariates, but was no longer significant after adding the additional covariates concerning health-behavioral characteristics and current stress (see **Supplementary Table 4A**).

**Table 4:** Logistic regression analyses on the association between adverse cardiovascular outcome (aCVO) and xBRS and heart rate variability.

	R^2^	β	Odds Ratio	95% Confidence Interval	p
**xBRS**					
Model 1	0.041	−0.882	0.414	[0.356, 0.470]	<0.001
Model 2*	0.118	−0.534	0.586	[0.499, 0.689]	<0.001
Model 3*^†^	0.135	−0.209	0.811	[0.673, 0.978]	0.028
Model 4*^†‡^	0.192	−0.071	0.932	[0.769, 1.129]	0.470
**RMSSD**					
Model 1	0.018	−0.652	0.521	[0.441, 0.616]	<0.001
Model 2*	0.110	−0.360	0.698	[0.588, 0.828]	<0.001
Model 3*^†^	0.179	0.015	1.015	[0.844, 1.220]	0.877
Model 4*^†‡^	0.197	0.011	1.011	[0.838, 1.220]	0.909
**SDNN**					
Model 1	0.022	−0.868	0.420	[0.344, 0.513]	<0.001
Model 2*	0.111	−0.442	0.643	[0.524, 0.789]	<0.001
Model 3*^†^	0.179	−0.117	0.890	[0.717, 1.103]	0.286
Model 4*^†‡^	0.197	−0.080	0.923	[0.741, 1.150]	0.476

Each model shows the regression results of xBRS, RMSSD, and SDNN.

*Adjusted for antihypertensive medication.

^†^Adjusted for sociodemographic covariates (sex, age, education, and ethnicity).

^‡^Adjusted for health-behavioral and psychosocial covariates (smoking, alcohol, body mass index, physical activity, current stress).

xBRS, baroreflex sensitivity; RMSSD, a parameter reflecting heart rate variability calculated as the square root of the mean squared successive differences between adjacent normal-to-normal interbeat intervals; SDNN, a parameter reflecting heart rate variability calculated as the standard deviation of normal-to-normal interbeat intervals.

## Discussion

In this large, population-based, multi-ethnic urban cohort study, we tested whether exposure to child maltreatment as a specific form of direct ACEs is associated with higher risk of self-reported history of CVD and autonomic regulation as CVD risk factor. This is to our knowledge, the first study on the association between child maltreatment and CVD risk in a representative urban population: heterogeneous and generalizable on demographic factors such as SES, educational level, and ethnicity (29, 30).

Our study confirmed that child maltreatment is significantly associated with higher risk for CVD later in life. This association remained significant after adjusting for potentially relevant covariates. With every additional child maltreatment type reported, the odds for reporting aCVO was 7.8% (95% CI: 1.018–1.142) higher. Thus, child maltreatment was associated with an increased risk for adult aCVO over and above the effects of a range of sociodemographic, health-behavioral, and current stress factors, which were previously found to be associated with both increased self-reported ACEs and risk for CVD (3, 43, 44).

Our finding is in concordance with existing literature that ACEs are important determinants of health problems in adulthood and more specifically consistent with the results of previous studies examining the association between childhood maltreatment and CVD (39). These previous studies also reported a linear association between cumulative exposure and increased risk for self-reported CVD after adjustment for relevant covariates. However, this is the first study establishing this relationship in a heterogeneous, multi-ethnic, and thus Western urban population representative cohort as the HELIUS cohort.

In addition to self-reported history of CVD, we also examined the association between child maltreatment and autonomic regulation as CVD risk factor. We did find, as we expected, that the cumulative exposure to child maltreatment was negatively associated with autonomic regulation within models without any covariates added. However, this association disappeared after adding the sociodemographic covariates to the models, which in themselves were previously found to be associated with increased risk for ACEs and CVD (5, 12–16, 39). In agreement with our findings, some previous studies also did not find a significant direct association between HRV and ACEs. The study by van Ockenburg et al. (25), based on a randomly selected large cohort of people with albuminuria, found significantly lower HRV in individuals reporting ACEs, but as in the current study this association also disappeared after correcting for sociodemographic and health behavioral covariates (25). Winzeler et al. (24) found an association between ACEs and HRV in young healthy women when the HRV was measured during performance of a stress task and not during baseline measurements (24), which is in concordance with the null findings in our study, with HRV also measured during resting conditions.

Interestingly, to verify the assumed association between autonomic (dys) regulation and aCVO in our cohort, we also investigated associations between HRV, BRS, and aCVO. Contrary to our expectations, we observed that the initially observed negative associations between self-reported CVD and the objective measures of autonomic regulation were no longer present in the corrected models. Upon adding the potential sociodemographic confounders, the association between self-reported CVD and both HRV indices was no longer significant. Initially, we hypothesized that the absence of a stable association between HRV and CVD, which seems to be contradictory to the existing literature (19, 22), could be influenced by the composition of our cohort. First of all, as our cohort is relatively young and correspondingly aCVO prevalence is relatively low (5.2%), we may have had limited signal to detect these associations in the whole sample. However, we found that age was not a significant moderator in any of the associations. Secondly, the HELIUS study includes both individuals without prior CVD and individuals who have already experienced one or more CVD events, and thereby departs from the existing literature which investigated the association between autonomic regulation and future CVD events in either healthy populations or specific populations of CVD patients (19, 22, 45–53). However, a sensitivity analysis revealed that our results remained unchanged after excluding participants who reported CVD. The association between BRS and CVD remained significant upon adding the sociodemographic covariates, but was no longer significant upon additionally adding the health-behavioral and current stress factors, indicating that these factors may be mediating pathways influencing the formerly observed association between BRS and CVD (19).

Thus we did not observe an association between child maltreatment and autonomic regulation, nor an association between autonomic regulation and CVD after inclusion of covariates, although we cannot exclude that measuring these indices during specific physical or psychological challenges would reflect another pattern. Alternatively, ACEs including child maltreatment may trigger, besides ANS, a cascade of molecular alterations in other systems that regulate stress responses and may be involved in CVD development, such as neuroendocrine, immune systems, endothelial damage or accelerated atherosclerosis (54).

In addition, there may be psychological mechanisms that may also play a role in the association between ACEs, including child maltreatment, and increased CVD risk, such as maladaptive cognitive models, impaired attachment, dysfunctional coping behaviors, and unhealthy peer associations (55). Moreover, studies suggest that ACEs and especially child maltreatment could induce emotional problems, including depression, anxiety, and affective lability, and this could be associated with CVD risk in adulthood (26). Thus, poor mental health could either moderate or mediate associations between ACEs and health outcomes (56). Additionally, use of psychotropic medication may additionally influence these associations.

Furthermore, adversity is only one part of the equation regarding childhood environmental influences on future health. One could argue that in the face of adversity, neither disease nor resilience is a certain outcome. The presence of protective factors, particularly safe, stable, and nurturing relationships, can often mitigate the consequences of ACEs (57, 58). Also neighborhood greenness for example might buffer against the detrimental effects of stress by specifically promoting activity of the parasympathetic nervous system in restoring the body to a calm state after stress reactivity (59). However, these factors were not considered in the current study.

### Strengths and Limitations

There are several strengths and weaknesses of this study that need to be considered when interpreting our results. The first major strength of this study is that the study was conducted in a large cohort, which provides notable statistical power. A random sampling technique was used through the municipality register of Amsterdam, which guarantees a non-selective, community recruited general population study sample. It is unlikely that the relatively low response rate of 28% has led to selection bias, as analysis of non-responders within our cohort established that there was no difference in socioeconomic characteristics between participants and non-participants (30).

Second, with the prevalence rate of any type of child maltreatment (around 30%) being in line with that of a large Dutch study on its prevalence in the general population (31), the experiences of participants in this study are representative for the Dutch general adult population, and results of our study are thus likely generalizable.

Potential limitations and weaknesses of our study also require consideration. First, the HELIUS data are cross-sectional, this poses constraints on the directionally in the investigated associations, especially in the association between autonomic regulation and CVD. Furthermore, the measurement of CVD by self-report may have led to under-reporting or over-reporting when compared to direct assessment of CVD events, although previous studies have shown a high degree of specificity for self-reported CVD and stroke (60, 61).

Moreover, we measured four types of child maltreatment: emotional neglect, psychological abuse, physical abuse, and sexual abuse, which certainly does not cover the full spectrum of ACEs. Furthermore, only the number of endorsed types of child maltreatment were assessed, not the overall severity, frequency of distinct types of maltreatment, or perceived impact of these experiences. We also did not examine the developmental timing and chronicity of maltreatment, which may also have an influence on the associations we investigated. Recent evidence indicates a distinctive impact of childhood adversity type and timing on physical and mental health and neurobiological correlates in adulthood, supporting the notion of stress-sensitive periods in (organ) development in childhood (62).

In addition, since the assessment of child maltreatment was based on retrospective self-report, effects of memory biases cannot be excluded. Moreover, child maltreatment may also occur before children have the cognitive ability to remember such events. Evaluating retrospective self-report to assess childhood maltreatment, a study performed on retrospective recalls of sexual and physical abuse, as well as physical and emotional neglect, ascertained retrospective surveys to be sufficiently valid (63). In contrast, a recent meta-analysis and systematic review performed on the extent of agreement between retrospective and prospective measures of child maltreatment concluded that prospective and retrospective measures cannot be used interchangeably to study risk mechanisms and associations with health outcomes (64). They mention that “caution should be used in assuming that retrospective reports accurately represent experiences, rather than perceptions, interpretations or existential recollections.” Thus, ideally our study should be replicated in a prospective design.

Moreover, missing data in the child maltreatment questionnaire were significantly more frequent in every ethnicity compared to the Dutch ethnicity, which may be related to two factors. First, there could be a larger component of shame and taboo within some ethnicities to report these adverse experiences (65). Second, the ethnic differences may have resulted from the formulation of our items on child maltreatment: it is possible that some participants would have endorsed the objective explanation (e.g. being beaten during childhood), but did not agree on the following interpretation of that behavior as representative of maltreatment (e.g. having experienced physical abuse). However, we applied multiple imputation with auxiliary variables to deal with the impact of non-randomly missing data, which presumably resulted in a more accurate estimation of the relationship between maltreatment and our outcome variables (40).

Finally, although our analyses concerning BRS and HRV included over 10,000 participants, these analyses only included 46.3% of the sample. Due to logistic constraints concerning availability of equipment, continuous BP measurements were not performed for 28.1%. An additional 15.6% of participants were excluded from analyses upon data preprocessing. We observed several statistically albeit small significant differences between included and non-included participants, and therefore cannot exclude results were impacted by this selective subset. Also, BRS and HRV were only assessed once. As these measurements are highly dynamic within an individual (66), the assessment could be more valuable after repeated measurements. Moreover, HRV and BRS were only assessed in resting conditions. We can therefore not exclude that associations between child maltreatment and the sympathovagal balance would be present under stressful conditions, such as in the study by Winzeler et al. (24).

### Conclusion, Implications, and Future Directions

The use of this large, population urban population representative sample provides insight into the long-term correlates of child maltreatment. A positive association was established between cumulative ACEs in the form of child maltreatment and risk on aCVO, over and above the effect of relevant demographic, health, and psychological factors. The association between child maltreatment and autonomic regulation indices was no longer present after correcting for sociodemographic factors. The quality of research on this topic will be strengthened with prospective longitudinal studies starting in early age and continuing into old age, more expansive measurement of child maltreatment, and other ACE types as well as potential resiliency factors and direct and objective assessments of CVD events and assessment of dynamic autonomic regulation.

## Data Availability Statement

The datasets generated for this study are available on request to the corresponding author.

## Ethics Statement

The HELIUS study has been approved by the Ethical Review Board of the Academic Medical Center Amsterdam. The patients/participants provided their written informed consent to participate in this study.

## Author Contributions

MB, MZ, and AL contributed to the conception and design of the current study. MS and DC organized the database. MB and MZ performed the statistical analysis. MB wrote the first draft of the manuscript. MZ and AL wrote sections of the manuscript. All authors contributed to manuscript revision, and read and approved the submitted version.

## Funding

The Amsterdam UMC, location Academic Medical Center (AMC) of Amsterdam and the Public Health Service of Amsterdam (GGD Amsterdam) provided core financial support for HELIUS. The HELIUS study is also funded by research grants of the Dutch Heart Foundation (Hartstichting; grant no. 2010T084), the Netherlands Organization for Health Research and Development (ZonMw; grant no. 200500003), the European Integration Fund (EIF; grant no. 2013EIF013) and the European Union (Seventh Framework Programme, FP-7; grant no. 278901).

## Conflict of Interest

The authors declare that the research was conducted in the absence of any commercial or financial relationships that could be construed as a potential conflict of interest.

## References

[1] WHO Adverse childhood experiences international questionnaire (ACE-IQ).

[2] Gilbert LK Breiding MJ Merrick MT Thompson WW Ford DC Dhingra SS Childhood adversity and adult chronic disease: an update from ten states and the District of Columbia, 2010. Am J Prev Med (2015) 48:345–9. 10.1016/j.amepre.2014.09.006 25300735

[3] Felitti VJ Anda RF Nordenberg D Williamson DF Spitz AM Edwards V Relationship of childhood abuse and household dysfunction to many of the leading causes of death in adults: the adverse childhood experiences (ACE) study. Am J Prev Med (1998) 56:774–86. 10.1016/S0749-3797(98)00017-8 31104722

[4] Merrick MT Ford DC Ports KA Guinn AS Prevalence of adverse childhood experiences from the 2011-2014 behavioral risk factor surveillance system in 23 states. JAMA Pediatr (2018) 172:1038–44. 10.1001/jamapediatrics.2018.2537 PMC6248156 30242348

[5] Hughes K Bellis MA Hardcastle KA Sethi D Butchart A Mikton C The effect of multiple adverse childhood experiences on health: a systematic review and meta-analysis. Lancet Public Heal (2017) 2:e356–66. 10.1016/S2468-2667(17)30118-4 29253477

[6] Norman RE Byambaa M De R Butchart A Scott J Vos T The long-term health consequences of child physical abuse, emotional abuse, and neglect: a systematic review and meta-analysis. PLoS Med (2012) 9. 10.1371/journal.pmed.1001349 PMC3507962 23209385

[7] Danese A McEwen BS Adverse childhood experiences, allostasis, allostatic load, and age-related disease. Physiol Behav (2012) 106:29–39. 10.1016/j.physbeh.2011.08.019 21888923

[8] Pechtel P Pizzagalli DA Effects of early life stress on cognitive and affective function: an integrated review of human literature. Psychopharmacology (2011) 214:55–79. 10.1007/s00213-010-2009-2 20865251 PMC3050094

[9] Anda RF Croft JB Felitti VJ Nordenberg D Giles WH Williamson DF Adverse childhood experiences and smoking during adolescence and adulthood. J Am Med Assoc (1999) 282:1652–8. 10.1001/jama.282.17.1652 10553792

[10] Bellis MA Lowey H Leckenby N Hughes K Harrison D Adverse childhood experiences: retrospective study to determine their impact on adult health behaviours and health outcomes in a UK population. J Public Heal (U K) (2014) 36:81–91. 10.1093/pubmed/fdt038 23587573

[11] Ford ES Anda RF Edwards VJ Perry GS Zhao G Li C Adverse childhood experiences and smoking status in five states. Prev Med (Baltim) (2011) 53:188–93. 10.1016/j.ypmed.2011.06.015 21726575

[12] Midei AJ Matthews KA Interpersonal violence in childhood as a risk factor for obesity: a systematic review of the literature and proposed pathways. Obes Rev (2011) 12:e159–72. 10.1111/j.1467-789X.2010.00823.x PMC3104728 21401850

[13] Danese A Tan M Childhood maltreatment and obesity: systematic review and meta-analysis. Mol Psychiatry (2014) 19:544–54. 10.1038/mp.2013.54 23689533

[14] Al Odhayani A Watson WJ Watson L Behavioural consequences of child abuse. Can Fam Physician (2013) 59:831–6 PMC3743691 23946022

[15] Metzler M Merrick MT Klevens J Ports KA Ford DC Adverse childhood experiences and life opportunities: shifting the narrative. Child Youth Serv Rev (2017) 72:141–9. 10.1016/j.childyouth.2016.10.021 PMC10642285 37961044

[16] Benjamin EJ Virani SS Callaway CW Chamberlain AM Chang AR Cheng S Heart disease and stroke statistics - 2018 update: a report from the american heart association. Circulation (2018) 137:e67. 10.1161/CIR.0000000000000558. 29386200

[17] Swenne CA Baroreflex sensitivity: mechanisms and measurement. Netherlands Hear J (2013) 21:58–60. 10.1007/s12471-012-0346-y PMC3547418 23179611

[18] Kleiger RE Stein PK Bigger JT Heart rate variability: measurement and clinical utility. Ann Noninvasive Electrocardiol (2005) 10:88–101. 10.1111/j.1542-474X.2005.10101.x 15649244 PMC6932537

[19] La Rovere MT Bigger JT Marcus FI Mortara A Schwartz PJ Baroreflex sensitivity and heart-rate variability in prediction of total cardiac mortality after myocardial infarction. ATRAMI (Autonomic Tone and Reflexes After Myocardial Infarction) investigators. Lancet (1998a). 351:478–84. 10.1016/S0140-6736(97)11144-8 9482439

[20] VanRavenswaaij-Arts C Koll´ee L Hopman J Stoelinga G VanGeijn H Heart rate variability, review. Ann Intern Med (1993) 118:436–47. 10.7326/0003-4819-118-6-199303150-00008 8439119

[21] Bigger JT Fleiss JL Steinman RC Rolnitzky LM Kleiger RE Rottman JN Frequency domain measures of heart period variability and mortality after myocardial infarction. Circulation (1992) 85:164–71. 10.1161/01.CIR.85.1.164 1728446

[22] Huikuri HV Stein PK Heart rate variability in risk stratification of cardiac patients. Prog Cardiovasc Dis (2013) 56:153–9. 10.1016/j.pcad.2013.07.003 24215747

[23] McEwen BS Stellar E Stress and the individual: mechanisms leading to disease. Arch Intern Med (1993) 153:2093–101. 10.1001/archinte.1993.00410180039004 8379800

[24] Winzeler K Voellmin A Hug E Kirmse U Helmig S Princip M Adverse childhood experiences and autonomic regulation in response to acute stress: the role of the sympathetic and parasympathetic nervous systems. Anxiety Stress Coping (2017) 30:145–54. 10.1080/10615806.2016.1238076 27653030

[25] van Ockenburg SL Tak LM Bakker SJL Gans ROB de Jonge P Rosmalen JGM Effects of adverse life events on heart rate variability, cortisol, and C-reactive protein. Acta Psychiatr Scand (2015) 131:40–50. 10.1111/acps.12286 24833194

[26] Jin MJ Kim JS Kim S Hyun MH Lee SH An integrated model of emotional problems, beta power of electroencephalography, and low frequency of heart rate variability after childhood trauma in a non-clinical sample: a path analysis study. Front Psychiatry (2018) 8:314. 10.3389/fpsyt.2017.00314 PMC5786859 29403401

[27] Stone LB Amole MC Cyranowski JM Swartz HA History of childhood emotional abuse predicts lower resting-state high-frequency heart rate variability in depressed women. Psychiatry Res (2018) 269:681–7. 10.1016/j.psychres.2018.08.106 PMC6223021 30273892

[28] Miskovic V Schmidt LA Georgiades K Boyle M MacMillan HL Stability of resting frontal electroencephalogram (EEG) asymmetry and cardiac vagal tone in adolescent females exposed to child maltreatment. Dev Psychobiol (2009) 51:474–87. 10.1002/dev.20387 19629997

[29] Stronks K Snijder MB Peters RJ Prins M Schene AH Zwinderman AH Unravelling the impact of ethnicity on health in Europe: the HELIUS study. BMC Public Health (2013) 13:402. 10.1186/1471-2458-13-402 PMC3646682 23621920

[30] Snijder MB Galenkamp H Prins M Derks EM Peters RJG Zwinderman AH Cohort profile: the Healthy Life in an Urban Setting (HELIUS) study in Amsterdam, the Netherlands. BMJ Open (2017) 7. 10.1136/bmjopen-2017-017873 PMC5736025 29247091

[31] De Graaf RON Ten Have M Van Dorsselaer S The Netherlands mental health survey and incidence study-2 (NEMESIS-2): design and methods. Int J Methods Psychiatr Res (2010) 33:581–6. 10.1002/mpr.317 PMC6878518 20641046

[32] van Nieuwenhuizen B Collard D Tan H Blom M van den Born B-J Kunst A Socioeconomic differences in vagal activity: the HELIUS study. Submitted. 10.1097/PSY.0000000000000887 33196631

[33] Task Force of the European Society of Cardiology the North American Society of Pacing Electrophysiology Standards of measurement, physiological interpretation, and clinical use. Circulation (1996) 93:1043–65. 8598068

[34] Malik M Camm AJ Bigger JT Breithardt G Cerutti S Cohen RJ Heart rate variability. Standards of measurement, physiological interpretation, and clinical use. Eur Heart J (1996) 1:151–81. 10.1093/oxfordjournals.eurheartj.a014868

[35] Shaffer F Ginsberg JP An overview of heart rate variability metrics and norms. Front Public Heal (2017) 5:258. 10.3389/fpubh.2017.00258 PMC5624990 29034226

[36] Shaffer F McCraty R Zerr CL A healthy heart is not a metronome: an integrative review of the heart's anatomy and heart rate variability. Front Psychol (2014) 5:1040. 10.3389/fpsyg.2014.01040 PMC4179748 25324790

[37] Wesseling KH Karemaker JM Castiglioni P Toader E Cividjian A Settels JJ Validity and variability of xBRS: instantaneous cardiac baroreflex sensitivity. Physiol Rep (2017) 5:e13509. 10.14814/phy2.13509 PMC5704083 29180481

[38] Westerhof BE Gisolf J Stok WJ Wesseling A KH Karemaker JM Time-domain cross-correlation baroreflex sensitivity: performance on the EUROBAVAR data set. J Hypertens (2004) 22:1371–90. 10.1097/01.hjh.0000125439.28861.ed 15201554

[39] Basu A McLaughlin KA Misra S Koenen KC Childhood maltreatment and health impact: the examples of cardiovascular disease and type 2 diabetes mellitus in adults. Clin Psychol Sci Pract (2017) 24(2):125–39. 10.1111/cpsp.12191 PMC5578408 28867878

[40] Asendorpf JB Van De Schoot R Denissen JJA Hutteman R Reducing bias due to systematic attrition in longitudinal studies: the benefits of multiple imputation. Int J Behav Dev (2014) 38:453–60. 10.1177/0165025414542713

[41] Archer G Pinto Pereira S Power C Child maltreatment as a predictor of adult physical functioning in a prospective British birth cohort. BMJ Open (2017) 7:e017900. 10.1136/bmjopen-2017-017900 PMC5665268 29079607

[42] Stuart EA Azur M Frangakis C Leaf P Multiple imputation with large data sets: a case study of the children's mental health initiative. Am J Epidemiol (2009) 169:1133–9. 10.1093/aje/kwp026 PMC2727238 19318618

[43] Dong M Giles WH Felitti VJ Dube SR Williams JE Chapman DP Insights into causal pathways for ischemic heart disease: adverse childhood experiences study. Circulation (2004) 110:1761–6. 10.1161/01.CIR.0000143074.54995.7F 15381652

[44] Korkeila J Vahtera J Korkeila K Kivimäki M Sumanen M Koskenvuo K Childhood adversities as predictors of incident coronary heart disease and cerebrovascular disease. Heart (2010) 96:298–303. 10.1136/hrt.2009.188250 20194205

[45] Stein PK Domitrovich PP Huikuri HV Kleiger RE Traditional and nonlinear heart rate variability are each independently associated with mortality after myocardial infarction. J Cardiovasc Electrophysiol (2005) 16:13–20. 10.1046/j.1540-8167.2005.04358.x 15673380

[46] Perkiömäki JS Jokinen V Tapanainen J Airaksinen KEJ Huikuri HV Autonomic markers as predictors of nonfatal acute coronary events after myocardial infarction. Ann Noninvasive Electrocardiol (2008) 13:120–9. 10.1111/j.1542-474X.2008.00211.x PMC6932524 18426437

[47] Kleiger RE Miller JP Bigger JT Moss AJ Decreased heart rate variability and its association with increased mortality after acute myocardial infarction. Am J Cardiol (1987) 59:256–62. 10.1016/0002-9149(87)90795-8 3812275

[48] Malik M Farrell T Cripps T Camm AJ Heart rate variability in relation to prognosis after myocardial infarction: selection of optimal processing techniques. Eur Heart J (1989) 10:1060–74. 10.1093/oxfordjournals.eurheartj.a059428 2606116

[49] Kubota Y Chen LY Whitsel EA Folsom AR Heart rate variability and lifetime risk of cardiovascular disease: the atherosclerosis risk in communities study. Ann Epidemiol (2017) 27:619–25. 10.1016/j.annepidem.2017.08.024 PMC5821272 29033120

[50] Farrell TG Paul V Cripps TR Malik M Bennett ED Ward D Baroreflex sensitivity and electrophysiological correlates in patients after acute myocardial infarction. Circulation (1991) 83:945–52. 10.1161/01.CIR.83.3.945 1999042

[51] Farrell TG Odemuyiwa O Bashir Y Cripps TR Malik M Ward DE Prognostic value of baroreflex sensitivity testing after acute myocardial infarction. Br Heart J (1992) 67:129–37. 10.1136/hrt.67.2.129 PMC1024741 1540432

[52] La Rovere MT Specchia G Mortara A Schwartz PJ Baroreflex sensitivity, clinical correlates, and cardiovascular mortality among patients with a first myocardial infarction: a prospective study. Circulation (1988c). 78:816–24. 10.1161/01.CIR.78.4.816 3168190

[53] Schwartz PJ Zaza A Pala M Locati E Beria G Zanchetti A Baroreflex sensitivity and its evolution during the first year after myocardial infarction. J Am Coll Cardiol (1988) 12:629–36. 10.1016/S0735-1097(88)80048-2 3403820

[54] Su S Wang X Kapuku GK Treiber FA Pollock DM Harshfield GA Adverse childhood experiences are associated with detrimental hemodynamics and elevated circulating endothelin-1 in adolescents and young adults. Hypertension (2014) 64:201–7. 10.1161/HYPERTENSIONAHA.113.02755 PMC4057352 24777980

[55] Kendall-Tackett K The health effects of childhood abuse: four pathways by which abuse can influence health. Child Abus Negl (2002) 26:715–29. 10.1016/S0145-2134(02)00343-5 12201164

[56] Monnat SM Chandler RF Long-term physical health consequences of adverse childhood experiences. Sociol Q (2015) 56:723–52. 10.1111/tsq.12107 PMC4617302 26500379

[57] Masten AS Global perspectives on resilience in children and youth. Child Dev (2014) 85:6–20. 10.1111/cdev.12205 24341286

[58] Larkin H Shields JJ Anda RF The health and social consequences of adverse childhood experiences (ACE) across the lifespan: an introduction to prevention and intervention in the community. J Prev Interv Commun (2012) 40:263–70. 10.1080/10852352.2012.707439 22970779

[59] van den Berg MMHE Maas J Muller R Braun A Kaandorp W van Lien R Autonomic nervous system responses to viewing green and built settings: differentiating between sympathetic and parasympathetic activity. Int J Environ Res Public Health (2015) 12:15860–74. 10.3390/ijerph121215026 PMC4690962 26694426

[60] Bergmann MM Byers T Freedman DS Mokdad A Validity of self-reported diagnoses leading to hospitalization: a comparison of self-reports with hospital records in a prospective study of American adults. Am J Epidemiol (1998) 147:969–77. 10.1093/oxfordjournals.aje.a009387 9596475

[61] Yamagishi K Ikeda A Iso H Inoue M Tsugane S Self-reported stroke and myocardial infarction had adequate sensitivity in a population-based prospective study JPHC (Japan Public Health Center)-based Prospective Study. J Clin Epidemiol (2009) 62:667–73. 10.1016/j.jclinepi.2008.07.016 19108984

[62] Pechtel P Lyons-Ruth K Anderson CM Teicher MH Sensitive periods of amygdala development: the role of maltreatment in preadolescence. Neuroimage (2014) 97:236–44. 10.1016/j.neuroimage.2014.04.025 PMC4258391 24736182

[63] Brewin CR Andrews B. Gotlib IH Psychopathology and early experience: a reappraisal of retrospective reports. Psychol Bull (1993) 113(1);82. 8426875 10.1037/0033-2909.113.1.82

[64] Baldwin JR Reuben A Newbury JB Danese A Agreement between prospective and retrospective measures of childhood maltreatment. JAMA Psychiatry (2019) 76:584–93. 10.1001/jamapsychiatry.2019.0097 PMC6551848 30892562

[65] Glück TM Knefel M Lueger-Schuster B A network analysis of anger, shame, proposed ICD-11 post-traumatic stress disorder, and different types of childhood trauma in foster care settings in a sample of adult survivors. Eur J Psychotraumatol (2017) 8:1372543 . 10.1080/20008198.2017.1372543 PMC5632767 29038691

[66] Kobayashi H Inter- and intra-individual variations of heart rate variability in japanese males. J Physiol Anthropol (2007) 26:173–7 . 10.2114/jpa2.26.173 17435361

