# Supplementary material for: Associations Between Child Maltreatment, Autonomic Regulation, and Adverse Cardiovascular Outcome in an Urban Population: The HELIUS Study
Source: Front Psychiatry. 2020 Mar 17;11:69. doi: 10.3389/fpsyt.2020.00069 (PMC7092011; doi:10.3389/fpsyt.2020.00069)
Supplement: Supplementary file 1 [file DataSheet_1.docx]

**Table 1. Differences in characteristics between participants included versus not-included in the analyses concerning autonomic regulation.**

|  | **Participants included in ANS analyses (N=10260)** | **Participants not included in ANS analyses (N=11905)** |
| --- | --- | --- |
| **Socio-demographics**  Age  Female  Educational level  No-lower  Intermediate-higher | 44.00 ± 13.10  5558 (54.2%)  4626 (45.5%)  5552 (54.5%) | 44.63 ± 13.28***  7252 (60.9%)***  5053 (42.9%)*** 6727 (57.1%) |
| **Health behaviour**  Smoking  Drinking alcohol  BMI | 2510 (24.5%)  5165 (50.6%)  27.55 ± 5.25 | 2792 (23.6%)  6056 (51.2%)  26.70 ± 5.27*** |
| **Child maltreatment**  Nr of types experienced  0  1  2  3  4 | 6757 (65.8%)  1627 (15.9%)  911 (8.9%)  737 (7.2%)  228 (2.2%) | 7733 (65.0%)*  1838 (15.4%)  1089 (9.1%)  903 (7.6%)  343 (2.9%) |
| **CVD** | 493 (4.9%) | 642 (5.5%)* |
|  |  |  |

Continuous data are described as means and standard deviations and group differences were tested using t-tests. Categorical data are described as frequency and percentage and group differences were tested using Chi-square tests. * p<.05, *** p<.001.

**Table 2. Logistic regression predicting aCVO. Full model.**

|  | **β** | **SE** | **OR** | **95% CI** | **p** | |
| --- | --- | --- | --- | --- | --- | --- |
| **Child maltreatment** |  |  |  |  |  |  |
| Nr of types | 0.075 | 0.029 | 1.078 | [1.018, 1.142] | 0.011 |  |
| **Socio-demographic** |  |  |  |  |  |  |
| Sex (male=ref) | -0.669 | 0.070 | 0.512 | [0.447, 0.587] | <0.001 |  |
| Age | 0.076 | 0.004 | 1.079 | [1.075, 1.083] | <0.001 |  |
| Education (no-low=ref) | -0.367 | 0.074 | 0.693 | [0.600, 0.799] | <0.001 |  |
| **Ethnicity (Dutch = ref)** |  |  |  |  |  |  |
| SA Surinamese | 0.745 | 0.114 | 2.106 | [1.880, 2.360] | <0.001 |  |
| Afr. Surinamese | 0.010 | 0.115 | 1.010 | [0.901, 1.133] | 0.928 |  |
| Ghanaian | 0.188 | 0.145 | 1.203 | [1.041, 1.391] | 0.202 |  |
| Turkish | 0.541 | 0.132 | 1.696 | [1.486, 1.935] | 0.000 |  |
| Moroccan | -0.178 | 0.146 | 0.820 | [0.708, 0.949] | 0.175 |  |
| Other | -0.242 | 0.254 | 0.765 | [0.593, 0.986] | 0.292 |  |
| **Health behaviour** |  |  |  |  |  |  |
| Roken (no=ref) | 0.460 | 0.075 | 1.556 | [1.342, 1.804] | <0.001 |  |
| Alcohol (no=ref) | -0.346 | 0.078 | 0.706 | [0.607, 0.820] | <0.001 |  |
| BMI | 0.033 | 0.006 | 1.032 | [1.021, 1.044] | <0.001 |  |
| Physical activity | -0.251 | 0.068 | 0.780 | [0.689, 0.882] | <0.001 |  |
| **Psychological** |  |  |  |  |  |  |
| Current stress | 0.383 | 0.075 | 1.467 | [1.267, 1.699] | <0.001 |  |
| Constant | -7.230 | 0.296 | 0.001 | [0.000, 0.001] | <0.001 |  |

Adjusted for antihypertensive medication, for socio-demographic covariates (gender, age, ethnicity, education), for health-behavioural and psychological covariates (smoking status, alcohol, BMI, physical activity, current stress).

**Table 3a. Multiple linear regression on association Child maltreatment and BRS.**

|  |  | **BRS** |  |
| --- | --- | --- | --- |
|  | β | 95% CI | p |
| **Child maltreatment** |  |  |  |
| Nr of types | -0.001 | [-0.007, 0.008] | 0.981 |
| **Socio-demographic** |  |  |  |
| Sex (male=ref) | 0.060 | [0.041, 0.080] | <0.001 |
| Age | -0.026 | [-0.026,-0.025] | <0.001 |
| Education (no-low=ref) | 0.061 | [0.050, 0.072] | <0.001 |
| **Ethnicity (Dutch = ref)** |  |  |  |
| SA Surinamese | -0.099 | [-0.117,-0.081] | <0.001 |
| Afr. Surinamese | 0.035 | [0.019,0.051] | 0.027 |
| Ghanaian | 0.002 | [-0.022, 0.027] | 0.987 |
| Turkish | -0.144 | [-0.164,-0.125] | <0.001 |
| Moroccan | -0.065 | [-0.088,-0.042] | 0.007 |
| **Health behaviour** |  |  |  |
| Smoking (no=ref) | -0.012 | [-0.035, 0.010] | 0.299 |
| Alcohol (no=ref) | 0.022 | [-0.004,0.047] | 0.083 |
| BMI | -0.018 | [-0.020,-0.017] | <0.001 |
| Phys. activity (no=ref) | 0.081 | [0.071, -0.091] | <0.001 |
| **Psychological** |  |  |  |
| Current stress | 0.003 | [-0.017,-0.024] | 0.747 |
| Antihypertensives (no=ref) | -0.052 | [-0.066,-0.037] | <0.001 |
| Constant | 3.850 | [3.765,3.935] | <0.001 |
|  |  |  |  |

Adjusted for antihypertensive medication, for socio-demographic covariates (gender, age, ethnicity, education), for health-behavioural and psychological covariates (smoking status, alcohol, BMI, physical activity, current stress).

**Table 3b. Multiple linear regression on association Child maltreatment and HRV. Full model.**

|  |  | **RMSSD** |  |  | **SDNN** |  |
| --- | --- | --- | --- | --- | --- | --- |
|  | β | 95% CI | p | β | 95% CI | p |
| Child maltreatment |  |  |  |  |  |  |
| Nr of types | -0.007 | [-0.016, 0.003] | 0.169 | 0.001 | [-0.007, 0.009] | 0.747 |
| Socio-demographic |  |  |  |  |  |  |
| Sex (male=ref) | 0.098 | [0.077,0.118] | <0.001 | -0.080 | [-0.025, 0.009] | 0.366 |
| Age | -0.019 | [-0.02, -0.018] | <0.001 | -0.012 | [-0.013,-0.012] | <0.001 |
| Education (no-low=ref) | 0.021 | [0.006, 0.035] | 0.068 | 0.047 | [0.032, 0.062] | <0.001 |
| Ethnicity (Dutch = ref) |  |  |  |  |  |  |
| SA Surinamese | -0.086 | [-0.104,-0.067] | <0.001 | -0.081 | [-0.096,-0.065] | <0.001 |
| Afr. Surinamese | 0.017 | [0.001, 0.034] | 0.292 | -0.055 | [-0.069,-0.041] | <0.001 |
| Ghanaian | 0.051 | [0.031,0.071] | 0.011 | -0.066 | [-0.083,-0.049] | <0.001 |
| Turkish | -0.135 | [-0.155,-0.115] | <0.001 | -0.112 | [-0.129,-0.095] | <0.001 |
| Moroccan | -0.052 | [-0.072,-0.032] | 0.009 | -0.089 | [-0.106,-0.072] | <0.001 |
| Health behaviour |  |  |  |  |  |  |
| Roken (no=ref) | 0.068 | [0.044, 0.092] | <0.001 | 0.029 | [0.009, 0.049] | 0.005 |
| Alcohol (no=ref) | 0.015 | [-0.010, 0.039] | 0.253 | 0.025 | [0.005, 0.045] | 0.021 |
| BMI | -0.008 | [-0.009,-0.007] | <0.001 | -0.010 | [-0.011,-0.009] | <0.001 |
| Phys. activity (no=ref) | 0.086 | [0.075, 0.096] | <0.001 | 0.054 | [0.045,0.063] | <0.001 |
| Psychological |  |  |  |  |  |  |
| Current stress | 0.004 |  | 0.677 | -0.004 | [-0.022, 0.013] | 0.629 |
| Antihypertensives | -0.007 | [-0.034, 0.019] | 0.632 | -0.048 | [-0.070,-0.027] | <0.001 |
| Constant | 4.516 | [4.425,4.607] | <0.001 | 4.623 | [4.547, 4.698] | <0.001 |

Adjusted for antihypertensive medication, for socio-demographic covariates (gender, age, ethnicity, education), for health-behavioural and psychological covariates (smoking status, alcohol, BMI, physical activity, current stress) and for antihypertensive use.

**Table 3c. Sensitivity analysis on association child maltreatment and HRV/BRS – Full model.**

|  | R^2^ | β | 95% CI | p |
| --- | --- | --- | --- | --- |
| xBRS |  |  |  |  |
| Model 1 | 0.001 | -0.014 | [-0.027,-0.002] | 0.019 |
| Model 2 * | 0.063 | -0.016 | [-0.027,-0.004] | 0.009 |
| Model 3 *† | 0.371 | 0.001 | [-0.008, 0.011] | 0.781 |
| Model 4 *† ‡ | 0.396 | 0.000 | [-0.009, 0.010] | 0.922 |
| RMSSD |  |  |  |  |
| Model 1 | 0.001 | -0.015 | [-0.026,-0.004] | 0.009 |
| Model 2 * | 0.028 | -0.015 | [-0.026,-0.005] | 0.005 |
| Model 3 *† | 0.224 | -0.004 | [-0.014, 0.006] | 0.460 |
| Model 4 *† ‡ | 0.237 | -0.007 | [-0.017, 0.003] | 0.179 |
| SDNN |  |  |  |  |
| Model 1 | 0.000 | -0.005 | [-0.014,0.004] | 0.245 |
| Model 2 * | 0.038 | -0.006 | [-0.015, 0.003] | 0.186 |
| Model 3 *† | 0.181 | 0.003 | [-0.005,0.011] | 0.513 |
| Model 4 *† ‡ | 0.196 | 0.001 | [-0.007, 0.009] | 0.772 |

Each model shows the regression results of CHILD MALTREATMENT-number of types endorsed on xBRS/RMSSD and SDNN. *Adjusted for antihypertensive medication † Adjusted for socio-demographic covariates (sex, age, education and ethnicity), ‡Adjusted for health-behavioural and psychosocial covariates(smoking, alcohol, BMI, physical activity, current stress).

**Table 4a. Multiple linear regression association BRS and aCVO. Full model.**

|  |  | **BRS** |  |
| --- | --- | --- | --- |
|  | OR | 95% CI | p |
| **Parameter** |  |  |  |
| BRS | 0.945 | [0.779,1.146] | 0.567 |
| **Socio-demographic** |  |  |  |
| Sex (male=ref) | 0.503 | [0.406,0.623] | <0.001 |
| Age | 1.051 | [1.039,1.064] | <0.001 |
| Education (no-low=ref) | 0.780 | [0.607, 0.942] | 0.013 |
| Ethnicity (Dutch = ref) |  |  |  |
| SA Surinamese | 2.284 | [1.601,3.259] | <0.001 |
| Afr. Surinamese | 0.987 | [0.960,1.413] | 0.944 |
| Ghanaian | 1.293 | [0.852, 1.964] | 0.227 |
| Turkish | 2.081 | [1.391, 3.116] | <0.001 |
| Moroccan | 1.006 | [0.632, 1.602] | 0.980 |
| **Health behaviour** |  |  |  |
| Smoking (no=ref) | 1.922 | [1.537, 2.403] | <0.001 |
| Alcohol (no=ref) | 0.787 | [0.623,0.994] | 0.044 |
| BMI | 1.012 | [0.990,1.033] | 0.290 |
| Physical activity | 0.821 | [0.669, 1.006] | 0.057 |
| **Psychological** |  |  |  |
| Current stress | 1.441 | [1.151,1.803] | 0.001 |
| **Antihypertensives** | 3.930 | [3.143, 4.915] | <0.001 |
| Constant | 0.003 | [0.001,0.009] | <0.001 |

Adjusted for antihypertensive medication, for socio-demographic covariates (gender, age, ethnicity, education), for health-behavioural and psychological covariates (smoking status, alcohol, BMI, physical activity, current stress).

**Table 4b. Multiple linear regression association HRV and aCVO. Full model.**

|  | **RMSSD** |  |  |  | **SDNN** |  |
| --- | --- | --- | --- | --- | --- | --- |
|  | OR | 95% CI | p | OR | 95%CI | p |
| **Parameter** |  |  |  |  |  |  |
| RMSDD or SDNN | 1.011 | [0.838, 1.220] | 0.909 | 0.923 | [0.741, 1.150] | 0.476 |
| **Socio-demographic** |  |  |  |  |  |  |
| Sex (male=ref) | 0.501 | [0.404, 0.621] | <0.001 | 0.503 | [0.406, 0.623] | <0.001 |
| Age | 1.053 | [1.041, 1.065] | <0.001 | 1.052 | [1.040, 1.064] | <0.001 |
| Education (no-low=ref) | 0.754 | [0.605, 0.939] | 0.012 | 0.757 | [0.608, 0.943] | 0.013 |
| Ethnicity (Dutch = ref) |  |  |  |  |  |  |
| SA Surinamese | 2.291 | [1.606, 3.269] | <0.001 | 2.281 | [1.598, 3.254] | <0.001 |
| Afr. Surinamese | 0.980 | [0.685, 1.403] | 0.914 | 0.981 | [0.686, 1.403] | 0.915 |
| Ghanaian | 1.286 | [0.847, 1.953] | 0.238 | 1.286 | [0.847, 1.952] | 0.237 |
| Turkish | 2.095 | [1.400, 3.136] | <0.001 | 2.081 | [1.391, 3.115] | <0.001 |
| Moroccan | 1.006 | [0.632, 1.601] | 0.981 | 1.005 | [0.631, 1.601] | 0.982 |
| **Health behaviour** |  |  |  |  |  |  |
| Roken (nee=ref) | 1.921 | [1.536, 2.403] | <0.001 | 1.927 | [1.541, 2.410] | <0.001 |
| Alcohol (nee=ref) | 0.785 | [0.622, 0.991] | 0.042 | 0.788 | [0.624, 0.996] | 0.046 |
| BMI | 1.013 | [0.992, 1.034] | 0.242 | 1.012 | [0.991, 1.033] | 0.276 |
| Physical activity | 0.816 | [0.665- 1.000] | 0.050 | 0.820 | [0.669, 1.005] | 0.056 |
| **Psychological** |  |  |  |  |  |  |
| Current stress | 1.442 | [1.152, 1.804] | 0.001 | 1.439 | [1.150, 1.801] | 0.001 |
| **Antihypertensives** | 3.947 | [3.157, 4.935] | <0.001 | 3.924 | [3.137, 4.908] | <0.001 |
| Constant | 0.002 | [0.000, 0.005] | <0.001 | 0.002 | [0.001, 0.009] | <0.001 |

Adjusted for antihypertensive medication, for socio-demographic covariates (gender, age, ethnicity, education), for health-behavioural and psychological covariates (smoking status, alcohol, BMI, physical activity, current stress).

**Table 5. Interaction effects in association between child maltreatment and BRS.**

|  |  | **BRS** |  |
| --- | --- | --- | --- |
|  | β | 95% CI | p |
| **Interaction model gender** |  |  |  |
| *Reference = men* |  |  |  |
| child maltreatment x women | 0.000 | [-0.022,0.021] | 0.985 |
| **Interaction model age** |  |  |  |
| *Reference = age 54-70* |  |  |  |
| child maltreatment x 18-32 | -0.031 | [-0.059,-0.004] | 0.027 |
| child maltreatment x 33-45 | -0.007 | [-0.033, 0.019] | 0.601 |
| child maltreatment x 46-53 | -0.020 | [-0.045,0.006] | 0.133 |

Adjusted for antihypertensive medication, for socio-demographic covariates (gender, age, ethnicity, education), for health-behavioural and psychological covariates (smoking status, alcohol, BMI, physical activity, current stress).

**Table 5a. Interaction effects in association between child maltreatment and HRV.**

|  |  | **RMSSD** |  |  | **SDNN** |  |
| --- | --- | --- | --- | --- | --- | --- |
|  | β | 95% CI | p | β | 95% CI | p |
| **Interaction model gender** |  |  |  |  |  |  |
| *Reference = men* |  |  |  |  |  |  |
| child maltreatment x women | 0.008 | [-0.013,0.029] | 0.442 | 0.023 | [-0.015,0.018] | 0.864 |
| **Interaction model age** |  |  |  |  |  |  |
| *Reference = age 54-70* |  |  |  |  |  |  |
| child maltreatment x 18-32 | -0.014 | [-0.042, 0.015] | 0.348 | -0.012 | [-0.036, 0.011] | 0.307 |
| child maltreatment x 33-45 | 0.006 | [-0.020, 0.033] | 0.627 | -0.003 | [-0.018, 0.025] | 0.755 |
| child maltreatment x 46-53 | -0.010 | [-0.036, 0.016] | 0.457 | -0.007 | [-0.029,0.015] | 0.521 |

Adjusted for antihypertensive medication, for socio-demographic covariates (gender, age, ethnicity, education), for health-behavioural and psychological covariates (smoking status, alcohol, BMI, physical activity, current stress).

**Table 5b. Interaction effects in association between child maltreatment and aCVO.**

|  |  | **aCVO** |  |
| --- | --- | --- | --- |
|  | OR | 95% CI | p |
| **Interaction model gender** |  |  |  |
| *Reference = men* |  |  |  |
| child maltreatment x women | 1.104 | [0.927, 1.314] | 0.267 |
| **Interaction model age** |  |  |  |
| *Reference = age 54-70* |  |  |  |
| child maltreatment x 18-32 | 0.872 | [0.642, 1.184] | 0.380 |
| child maltreatment x 33-45 | 1.004 | [0.849, 1.188] | 0.960 |
| child maltreatment x 46-53 | 0.962 | [0.840, 1.101] | 0.571 |

Adjusted for antihypertensive medication, for socio-demographic covariates (gender, age, ethnicity, education), for health-behavioural and psychological covariates (smoking status, alcohol, BMI, physical activity, current stress).
